# Vitamin D and IFN-β Modulate the Inflammatory Gene Expression Program of Primary Human T Lymphocytes

**DOI:** 10.3389/fimmu.2020.566781

**Published:** 2020-12-04

**Authors:** Niccolò Bianchi, Stefan Emming, Chiara Zecca, Silvia Monticelli

**Affiliations:** ^1^ Institute for Research in Biomedicine (IRB), Università della Svizzera italiana (USI), Bellinzona, Switzerland; ^2^ Graduate School for Cellular and Biomedical Sciences, University of Bern, Bern, Switzerland; ^3^ Neurocenter of Southern Switzerland, Ospedale Regionale di Lugano, and Faculty of Biomedical Sciences, Università della Svizzera italiana, Lugano, Switzerland

**Keywords:** human T lymphocytes, vitamin D, IFN-β, gene expression, microRNAs, plasticity

## Abstract

IFN-β treatment is a commonly used therapy for relapsing-remitting multiple sclerosis (MS), while vitamin D deficiency correlates with an increased risk of MS and/or its activity. MS is a demyelinating chronic inflammatory disease of the central nervous system, in which activated T lymphocytes play a major role, and may represent direct targets of IFN-β and vitamin D activities. However, the underlying mechanism of action of vitamin D and IFN-β, alone or in combination, remains incompletely understood, especially when considering their direct effects on the ability of T lymphocytes to produce inflammatory cytokines. We profiled the expression of immune-related genes and microRNAs in primary human T lymphocytes in response to vitamin D and IFN-β, and we dissected the impact of these treatments on cytokine production and T cell proliferation. We found that the treatments influenced primarily memory T cell plasticity, rather than polarization toward a stable phenotype. Moreover, our data revealed extensive reprogramming of the transcriptional output of primary T cells in response to vitamin D and IFN-β and provide the bases for further mechanistic insights into these commonly used treatments.

## Introduction

Multiple sclerosis (MS) is an inflammatory disease of the central nervous system with an autoimmune etiology, in which self-reactive T lymphocytes are critically involved. Several T cell-derived inflammatory cytokines can impact the onset and severity of disease (1), with GM-CSF being a most prominent one. This is indicated, among other evidence, by the fact that mice lacking GM-CSF are resistant to the development of autoimmune inflammatory diseases, including experimental autoimmune encephalomyelitis (EAE), myocarditis, and collagen-induced arthritis (2–5). IFN-γ and IL-17 are also among the cytokines that can negatively affect MS disease or its animal model, EAE (1, 6). For instance, the percentage of IL-17-producing T_H_17 cells was expanded in MS patients and was shown to change with disease activity, increasing in patients with clinical exacerbation (7).

One of the commonly used treatment for MS patients is interferon-β (IFN-β), which was shown to reduce production of inflammatory cytokines by CD4^+^ T lymphocytes from relapsing-remitting MS patients (8). IFN-β can modulate cytokine production by T cells both directly and indirectly, by altering the phenotype of dendritic cells (DCs) (9, 10). Higher frequencies of GM-CSF-producing T cells were observed in MS patients compared to healthy controls, and these levels normalized upon IFN-β treatment (11). This treatment also led to increased IL-10 production, pointing toward a key role in dampening inflammatory responses in MS patients through multiple mechanisms. In the mouse, deletion of IFN-β or the IFN-α/β receptor (IFNAR) led to exacerbated EAE (12, 13), and deletion of IFNAR specifically in T regulatory (Treg) cells led to their defective responses, leaving them unable to control effector T cell activation and tissue inflammation (14). Vice versa, expression of IFNAR exclusively in the T cell compartment led to a milder course of EAE, pointing toward immune-modulatory roles of type I IFNs on T lymphocyte activity (15).

Several epidemiological, clinical, and immunological data also point toward a beneficial role of vitamin D in lowering MS risk and disease activity [reviewed in (16)]. *In vitro*, vitamin D treatment reduced GM-CSF expression (17), and increased the expression of FOXP3 and IL-10 (18, 19), suggesting that vitamin D is involved in restraining T cell responses. The effect of vitamin D on T cell proliferation remains instead somewhat controversial, with reports pointing either toward reduced proliferation (20) or no effect (19). An additional confounding factor when attempting to dissect the exact contribution of each of these treatments to T cell responses, is linked to the fact that IFN-β and vitamin D are often administered to MS patients in combination. Randomized clinical trials in relapsing-remitting MS patients did not report significant immune regulatory effects of high doses of vitamin D in MS patients (21–23), although the effect could have been at least partially masked by the concomitant IFN-β treatment. Indeed, whether vitamin D and IFN-β may have additive or synergistic effects remains controversial, with reports both in favor (24) and against (25) this possibility.

Overall, despite many observations pointing toward a crucial role of IFN-β and vitamin D in dampening inflammatory responses in MS, studies investigating a direct effect on T cell functions are often limited to the measurement of selected cytokines, or to the analysis of composite populations such as total peripheral blood mononuclear cells (PBMCs). Moreover, no information is available on the impact of the combined treatment (IFN-β + vitamin D) on gene and microRNA (miRNA) expression and T cell proliferation.

Here, we set out to systematically determine the changes in expression of immune-related genes and miRNAs in primary human T cell subsets treated with IFN-β and vitamin D, either alone or in combination, and we dissected the impact of these treatments on cytokine production and cell proliferation. We found that the treatments primarily modulated memory T cell plasticity, rather than inducing differentiation to a stable subset. Mechanistically, the two treatments differed substantially in their ability to modulate gene expression, with the effect of IFN-β being primarily linked to the induction of IL-10 and other anti-inflammatory genes, but also of *IL17* transcripts, while vitamin D had a more complex impact on gene expression. Both treatments also significantly impacted miRNA expression in T cells, most notably of miRNAs involved in the regulation of inflammatory responses and cell proliferation such as miR-155 and miR-150. Similar changes in miRNA and gene expression in response to treatments were observed in T lymphocytes from both healthy donors and MS patients, and also in the CD4^+^ and CD8^+^ subsets of T lymphocytes. Overall, our data provide a broad description of transcriptional changes occurring in human T lymphocytes in response to treatments frequently administered to MS patients.

## Materials and Methods

### Primary Human T Cell Isolation and Culture

Peripheral blood from healthy donors was obtained from the Swiss Blood Donation Center of Basel and Lugano (Switzerland), with informed consent from the Swiss Red Cross and authorization number CE 3428 from the Comitato Etico Canton Ticino. PBMCs were separated by gradient centrifugation (Ficoll-Paque Plus; GE Healthcare), followed by positive selection of CD4^+^ or CD8^+^ T lymphocytes using magnetic beads (Miltenyi Biotec). Naive and memory CD4^+^ T cell subsets were further separated using a FACSaria (BD Bioscience) as follows: naïve: CD4^+^CD25^–^CD45RA^+^CCR7^+^; central memory: CD4^+^CD25^–^CD45RA^–^CCR7^+^; effector memory: CD4^+^CD25^–^CD45RA^–^CCR7^–^. After magnetic separation, CD8^+^ T lymphocytes were sorted to exclude CD25^+^ cells and naïve T cells (CD45RA^+^CCR7^+^). The sorted cells were stimulated for 2 days with plate-bound recombinant anti-CD3 (clone TR66) and anti-CD28 (1 μg/ml) antibodies, followed by expansion in RPMI-1640 medium supplemented with 5% human serum, 1% non-essential amino acids, 1%, sodium pyruvate, 1% glutamine, penicillin, streptomycin, and 50 μM β-mercaptoethanol. After 48 h cells were removed from the stimuli and expanded for another 24–72 h without any further addition of cytokines to induce differentiation and/or proliferation. For vitamin D and IFN-β treatments, the culture medium was supplemented with vitamin D (100 nM 1α,25-dihydroxyvitamin D3; Sigma) and/or human IFN-β (2,500 U/ml; PeproTech) during the first plating and activation of the cells (day 0), and the treatments were not removed or re-added afterward. For experiments of T cell plasticity, T cell subsets were separated and treated as described above, except that in one group the culture medium was changed to remove vitamin D and IFN-β after 2 days of anti-CD3 and anti-CD28 stimulation, while in the second group the treatments were added only after the 2 days of stimulation. Analysis of the phenotype (cytokine production) was performed after 24–72h from the removal or addition of stimuli.

### Multiple Sclerosis Patients

Peripheral blood from three relapsing-remitting MS patients was obtained with informed consent and with approval of the Cantonal Ethics Committee (number CE TI 2906). All three patients were females between 32 and 43 years of age and not undergoing any treatment at the time of relapse, when peripheral blood was obtained (P1 and P3: treatment naïve; P2: discontinued IFN-β treatment 3 years earlier). Naïve and total memory T lymphocytes (CD4^+^CD25^–^CD45RA^–^CCR7^+/–^) were separated and treated as described above.

### Intracellular Cytokine Staining

CD4^+^ T cells were stimulated for 5h with PMA (phorbol 12-myristate 13-acetate, 200 nM) and ionomycin (1 µg/ml). For CD8^+^ T cells, 100 nM PMA and 0.5 µg/ml ionomycin were used. For the last 2.5 h of stimulation, brefeldin A (10 µg/ml) was added to the cells. After fixation (paraformaldehyde, 4%) and permeabilization [0.5% BSA and saponin in Dulbecco’s phosphate-buffered saline (DPBS)], the staining was performed with the directly conjugated anti-cytokine antibodies listed in **Supplementary Table 1**. For FOXP3 staining, a FOXP3 transcription factor staining kit (Thermo Fisher) was used, following manufacturer’s instructions. All samples were acquired on a Fortessa Flow Cytometer (BD Bioscience) and data was analyzed with FlowJo Software.

### Nanostring Sprint Profiling

For gene expression analysis, T cell samples were stimulated for 3 h with PMA and ionomycin prior to RNA extraction. Purified total RNA (50 ng) was hybridized to the nCounter Human Immunology v2 Panel codeset following exactly manufacturer’s instructions and analyzed using the Nanostring nCounter SPRINT profiler. After reads normalization, ratios and p-values were calculated with the nSolver 3.0 software. For miRNA expression analysis, 100 ng of total RNA were used in the Human v3 miRNA Assay. Data was normalized to spiked-in positive controls and to the 25 most highly expressed miRNAs.

### Quantitative RT-PCR

Total RNA was extracted using TRI reagent (MRC) and retrotranscribed using either the qScript cDNA SuperMix (Quanta Biosciences) for gene expression, or the TaqMan MicroRNA Reverse Transcription Kit (Applied Biosystems) for miRNA expression. Expression of *CSF2*, *IL10*, and *MYB* mRNAs was measured using the PerfeCTa SYBR Green FastMix (Quanta Bioscience) and the primers listed in **Supplementary Table 1**. For miRNA expression, the TaqMan probes (Applied Biosystems) listed in **Supplementary Table 1** were used. PCR reactions were run on the ABI 79000HT Fast Real-Time PCR System (Applied Biosystems) or a QuantStudio3 Real-Time PCR System (Thermo Fisher).

### Cell Proliferation

Cell proliferation was measured using an APC-BrdU Flow Kit from BD Biosciences, following the manufacturer’s instructions. Cells incorporated BrdU for 6 h at 37°C. Alternatively, human T lymphocytes were labeled with carboxyfluorescein succinimidyl ester (CFSE, 5 μM, Thermo Fischer Scientific) for 8 min at 37°C, and the dilution of the dye was recorded daily after cell activation.

### Statistical Analysis

Statistical analysis was performed with Prism software (GraphPad). Unless otherwise noted, data are represented as mean ± SD, and significance was determined by paired or unpaired Student’s t test, two-tailed.

## Results

### Vitamin D and IFN-β Modulate Memory T Cell Plasticity

**Figure 1:**
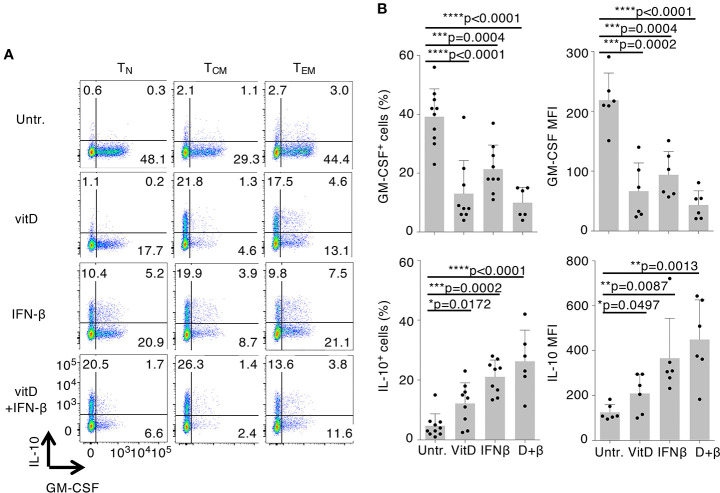
Vitamin D and IFN-β treatment modulate the inflammatory phenotype of CD4^+^ T lymphocytes. **(A)** Human CD4^+^ ****T_N_, T_CM_, and T_EM_ cells were activated with plate-bound anti-CD3 and anti-CD28 antibodies in the presence or absence of vitamin D and/or IFN-β. Intracellular staining for GM-CSF and IL-10 expression was performed after 5 days of culture using conjugated antibodies (IL-10-bv421, GM-CSF-PerCp-Cy5.5). **(B)** Compiled results of T_CM_ cells from different donors treated exactly as in a). Both the percentage of positive cells (left) and the mean fluorescence intensity (MFI, right) are shown. Each dot represents one donor (at least n=6). Mean ± SD; unpaired t-test, two-tailed, relative to untreated cells *p < 0.05; **p < 0.01; ***p < 0.001; ****p < 0.0001.

To investigate the immunomodulatory effects of vitamin D and IFN-β on cytokine production and gene expression, we first assessed the effect of these treatments on GM-CSF and IL-10 expression in different human T cell subpopulations (gating scheme in **Supplementary Figure 1A**). Naïve (T_N_), central memory (T_CM_), and effector memory (T_EM_) CD4^+^ cells isolated from peripheral blood of healthy donors were stimulated with plate-bound anti-CD3 and anti-CD28 antibodies and cultured for 5 days in the presence or absence of vitamin D and IFN-β, alone or in combination. Upon stimulation with PMA and ionomycin, both treatments led to diminished GM-CSF and increased IL-10 expression in all CD4^+^ T cell subsets, with T_CM_ cells being the most responsive to treatment and T_N_ being the most variable and least responsive subset (**Figure 1A** and **Supplementary Figure 1B**). This observation suggests that these experimental conditions influence primarily phenotypic plasticity of memory T cells (and especially T_CM_ cells, which retain higher plastic capacity), rather than differentiation of T_N_ cells (26). No cytokine expression was detected in the absence of T cell restimulation with PMA and ionomycin. Vitamin D significantly reduced GM-CSF expression in T_CM_ cells (**Figure 1B**). IFN-β was also able to reduce expression of GM-CSF, but to a lesser extent compared to vitamin D, and it primarily acted as a strong inducer of IL-10 expression. Interestingly, the combination of the two treatments did not further reduce GM-CSF expression, but enhanced IL-10 production. The effect of the treatments on IFN-γ was more modest, and somewhat reduced expression was observed only with vitamin D (**Supplementary Figure 1C**). A similar trend was observed also for IL-17 expression, although the percentage of IL-17-expressing cells was overall very small compared to the other cytokines (**Supplementary Figure 1C**).

**Figure 2:**
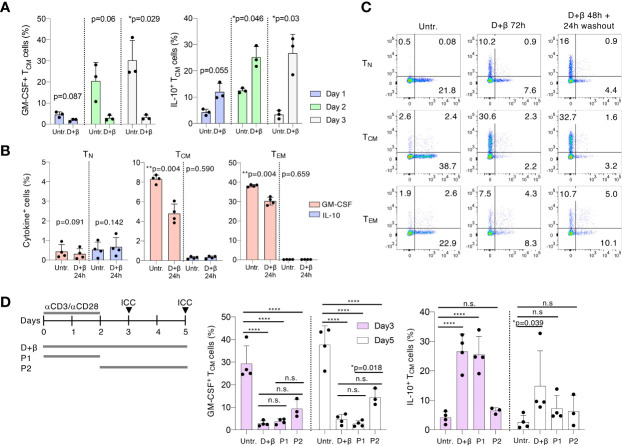
Vitamin D and IFN-β modulate memory T cell plasticity. **(A)** T_CM_ cells were freshly sorted from peripheral blood and were activated with plate-bound anti-CD3 and anti-CD28 antibodies in the presence or absence of vitamin D and IFN-β for the indicated times, followed by intracellular staining for GM-CSF and IL-10 expression. Each dot represents one donor (n=3). Mean ± SD; paired t-test, two-tailed, relative to untreated cells. **(B)** T cell subsets were freshly sorted from peripheral blood and were cultured for 24h with and without vitamin D and IFN-β prior to intracellular staining. Each dot represents one donor (n=4). Mean ± SD; paired t-test, two-tailed, relative to untreated cells. **(C)** T cell subsets were freshly sorted from peripheral blood and were activated with plate-bound anti-CD3 and anti-CD28 antibodies in the presence or absence of vitamin D and IFN-β for either three full days or for 2 days followed by a 24h washout. Expression of IL-10 and GM-CSF was measured by intracellular staining. Representative of n=4 donors. **(D)** Left: schematic representation of the experimental setup: T_CM_ cells were either left untreated or were treated with vitamin D and IFN-β continuously for 3 or 5 days; or were treated for 2 days followed by washout and culture for additional 24–72h (P1 condition); or the treatments were added at day 2 of stimulation for additional 24–72h (P2 condition). Middle and right panels: results of the intracellular staining (ICC, arrows in the schematic representation) at day 3 and day 5. Each dot represents one donor (n=3–4). Mean ± SD; two-way ANOVA. n.s. p > 0.05; *p < 0.05; **p < 0.01; ****p < 0.0001.

To better investigate the stability of the phenotype induced by the treatments, and to determine how rapidly it was established after stimulation, we activated freshly isolated T_CM_ cells with and without vitamin D and IFN-β and assessed cytokine expression at different time points. We found that a trend toward reduced GM-CSF and increased IL-10 expression was noticeable already after 24h of culture, and became significant between day 2 and day 3 of treatment, pointing toward early effects on the memory T cell phenotype (**Figure 2A**). Further highlighting a potential effect on memory T cell plasticity, treatment of freshly isolated, resting memory (T_CM_ and T_EM_) cells for 24h was already sufficient to reduce their ability to produce GM-CSF even in the absence of stimulation (**Figure 2B**). However, IL-10 production was not induced in cells not stimulated with anti-CD3 and anti-CD28 antibodies. In resting naïve T lymphocytes, the percentage of GM-CSF-producing cells remained low (<1%) in all conditions. These findings point toward a requirement for T cell activation to enhance IL-10 expression upon vitamin D and IFN-β treatment, but not for the loss of GM-CSF production. Next, we assessed the stability of the treatment-induced phenotype by activating the different T cell subsets in the presence of vitamin D and IFN-β for 2 days, followed by removal of the stimuli for 24h. We found that the reduced GM-CSF and increased IL-10 expression was maintained in all subsets even after removal of the stimuli, pointing toward a relative stability of the induced phenotype, at least short-term (**Figure 2C**). To further explore the effect of vitamin D and IFN-β on phenotype stability *vs.* T cell plasticity, we treated freshly sorted T_CM_ cells for either the initial 48h of anti-CD3 and anti-CD28 stimulation, followed by removal of the stimuli (P1 condition), or we added the treatments at day 2, followed by 24h or 72h of culture (P2 condition, **Figure 2D**). We found that the reduction in GM-CSF expression observed after the initial 48h of treatment was maintained even after 24 or 72h of washout (P1 conditions). Interestingly, treatment for 24h after the initial stimulation (P2 condition, day 3) was already sufficient to modulate GM-CSF expression in T_CM_ cells, pointing toward a rapid modulation of memory T cell plasticity. The reduction of GM-CSF expression was however somewhat less affected if the cells were treated after the initial 2 days of stimulation (P2 condition, day 5). After washout of the stimuli (P1 condition), expression of IL-10 was maintained for 24h, but not after 72h of washout, suggesting relatively lower stability of this phenotype compared to GM-CSF expression. Moreover, treatment of the cells after anti-CD3 and anti-CD28 stimulation, did not significantly increase IL-10 expression compared to cells treated during the activation stage (P2 condition, **Figure 2D**), suggesting that anti-CD3 and anti-CD28 stimulation is required for the enhancement of IL-10 expression mediated by the treatments. Overall, treatment of T cells with vitamin D and IFN-β modulated primarily memory T cell plasticity, leading to rapid and stable reduction in GM-CSF expression even in resting cells. Enhancement of IL-10 expression required instead concomitant TCR stimulation.

### Vitamin D and IFN-β Modulate Gene Expression in Human T Cell Subsets

**Figure 3:**
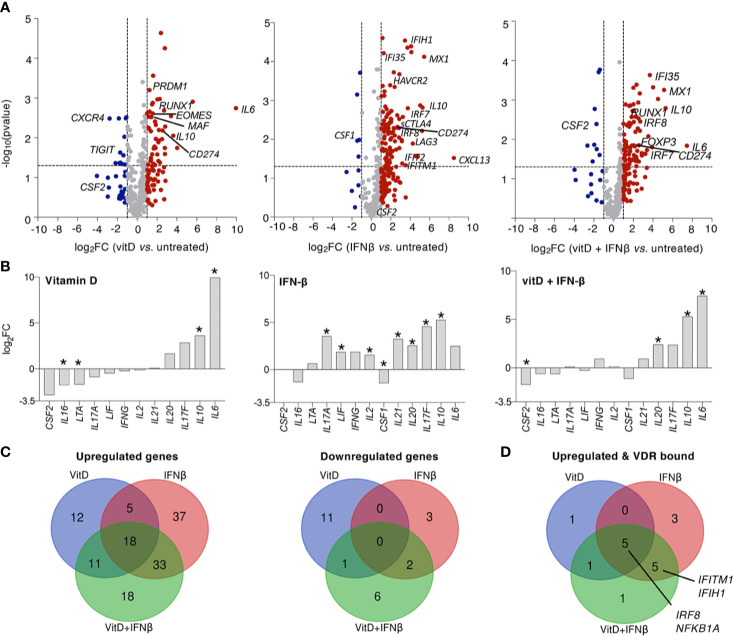
Vitamin D and IFN-β regulate the expression of immune transcripts in CD4^+^ T lymphocytes. **(A)** T_CM_ cells (2–5 independent donors) were treated for 5 days in the presence or absence of vitamin D and/or IFN-β and the expression of immune-relevant genes was determined by Nanostring profiling. **(B)** Same as **(A)**, but focusing on the expression of selected cytokines. The asterisk (*) indicates significant values (p<0.05). **(C)** Venn diagrams showing the extent of overlap between the different treatments. **(D)** Venn diagram showing the intersection between genes upregulated upon treatment and genes that were described to be bound directly by VDR by ChIP-seq (27).

To gain a better understanding of the effect of these two treatments on the expression of other cytokines and immune-related genes, we measured the expression of 570 transcripts by digital Nanostring profiling (**Supplementary Data Set 1**). We found that vitamin D treatment extensively affected the T cell transcriptional program, primarily by inducing the expression of several transcription factors known to impact T cell functions, such as *RUNX1*, but also by inducing the expression of immunomodulatory cytokines such as *IL10* and *IL6* (**Figure 3A**). The expression of several other cytokines was not significantly altered (**Figure 3B**). Overall, vitamin D altered the expression of a number of cytokines and factors, but many other inflammatory cytokine genes with potential roles in disease were unaffected. IFN-β treatment significantly induced the expression of a number of genes, including *IL10*, while very few genes were negatively regulated (**Figure 3C** and **Table 1**). Despite the anti-inflammatory factors induced by IFN-β (such as IL-10), this treatment also induced significant expression of both *IL17A* and *IL17F* transcripts. Finally, treatment of T cells with both vitamin D and IFN-β led to a combined phenotype, which shared features of both individual treatments, including a significant decrease in *CSF2* gene expression (encoding GM-CSF), together with significant upregulation of *IL10* expression, while the levels of *IL17* transcripts were less affected compared to IFN-β alone (**Figure 3B**). To better understand how many of the genes induced by vitamin D treatment could be directly regulated by the vitamin D receptor (VDR), we compared the list of genes induced by vitamin D in our dataset with the published list of genes that were bound and regulated by VDR (27). Using a log_2_FC>1 as threshold, we found an overlap of a few genes among the two datasets, which was largely similar when vitamin D was used alone or in combination with IFN-β (**Figure 3D**). Among these genes we identified not only *IRF8*, but also regulators of T cell activation and functionality, such as components of NF-kB signaling (*NFKBIA*). However, these genes were also induced after IFN-β treatment, pointing toward possible direct and indirect effects on these genes, or regulation mediated by both stimuli. We also searched the literature for STAT1 ChIP-seq data. We intersected our gene expression data with STAT1 ChIP-seq data (28) to search for putative IFN-β -regulated genes under the direct control of STAT1. As expected, we identified a number of IFN-inducible genes, including *MX1*, *IFI35* and *IRF7*, but also the *STAT1* and *STAT2* genes themselves, which were slightly upregulated, although post-transcriptional activation and hetero-dimerization with other STAT family members cannot be excluded (29).

**Table 1:** Genes up- or down-regulated by the treatments.

a. Upregulated genes		
Type of intersection	Number of genes	Gene names
IFNβ ∩ VitD ∩ VitD+IFNβ	18	*FKBP5 NFKBIA CTLA4_all SELPLG IFNGR1 CX3CR1 CTLA4-TM STAT4 TNFRSF8 SOCS3 IL6ST CD274 IL10 CCR5 sCTLA4 IL18RAP BCL3 BATF3*
IFNβ ∩ VitD	5	*HLA-C CEBPB ICAM1 MAPKAPK2 PRDM1*
IFNβ ∩ VitD+IFNβ	33	*XCL1 IFI35 ZAP70 CXCL10 LAMP3 TNFSF13B GBP1 STAT2 CTSS MX1 TRAF4 MYD88 IFITM1 TFRC IL18R1 IL20 GZMB IRF7 BST2 LAG3 TNFRSF13C NOTCH1 CASP1 NOD1 PYCARD SELL STAT5A BATF TAP2 IRF8 ICOS FAS CTSC*
VitD ∩ VitD+IFNβ	11	*LGALS3 CRADD MAP4K4 EOMES CD59 BCL2L11 IL6R MAF RUNX1 IL6 IL1RL1*
IFNβ	37	*IL17A PRF1 IL17F CD45R0 IL10RA IL21 SIGIRR BTLA CD6 STAT6 PML TRAF3 ATG16L1 CXCL13 LIF HAVCR2 CD27 CFB ICAM2 JAK2 NFIL3 TNFAIP3 GPR183 IKBKE TRAF1 STAT1 IFIH1 TRAF5 GZMA CD58 PTGER4 CD48 CD164 SLAMF7 CD97 IFIT2 IL2*
VitD	12	*RARRES3 IL1RAP HRAS ABL1 JAK3 CCL22 CD7 CTNNB1 SLC2A1 CD79A IL1R2 IGF2R*
VitD+IFNβ	18	*TRAF2 IRF4 ITGAM IDO1 STAT5B TNFSF8 IRAK4 IL2RA JAK1 MBP IL2RB ATG7 SPP1 ADA FOXP3 NFKB1 IKZF1 PSMB9*
**b. Downregulated genes**		
**Type of intersection**	**Number of genes**	**Gene names**
IFNβ ∩ VitD ∩ VitD+IFNβ	0	
IFNβ ∩ VitD	0	
IFNβ ∩ VitD+IFNβ	2	*HLA-DRB3 CD74*
VitD ∩ VitD+IFNβ	1	*GPI*
IFNβ	3	*DUSP4 CSF1 HLA-DRB*
VitD	11	*TIGIT PTPN6 EGR1 IFIT2 ICOSLG IL16 CD5 LTA ITGAL CXCR4 ITGA4*
VitD+IFNβ	6	*CCL22 HLA-DMA CSF2 HLA-DPB1 CD70 TMEM173*

**Figure 4:**
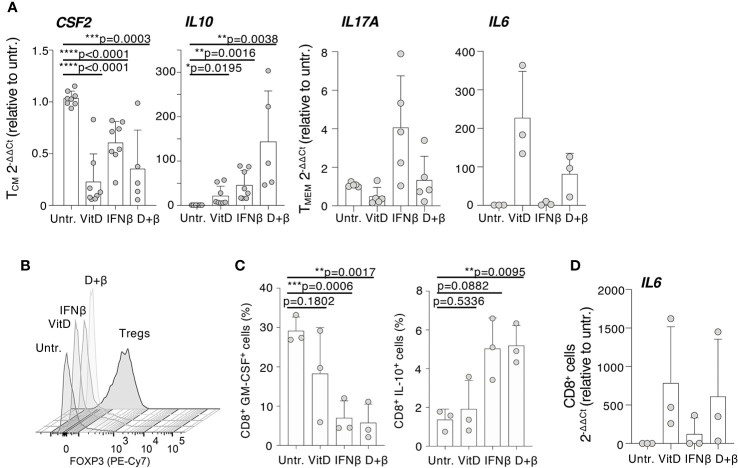
Modulation of specific immune transcripts in CD4^+^ and CD8^+^ T lymphocytes. **(A)** CD4^+^ T cells (either T_CM_ or total memory, T_MEM_) were activated with plate-bound anti-CD3 and anti-CD28 antibodies in the presence or absence of vitamin D and/or IFN-β. Expression of the indicated transcripts (*CSF2*, *IL10*, *IL17A*, and *IL6*) was determined by qRT-PCR. Each dot represents one donor (at least n=3). Mean ± SD; unpaired t-test, two-tailed, relative to untreated cells. **(B)** Memory CD4^+^ T lymphocytes were stimulated and treated with vitamin D and/or IFN-β. At day 5, expression of FOXP3 was determined by intracellular staining. Freshly isolated Treg cells were also used in comparison (representative of n=2 independent experiments). **(C)** Memory CD8^+^ T lymphocytes were stimulated and treated with vitamin D and/or IFN-β. After 5 days, the percentage of cells expressing the indicated cytokines was determined by intracellular staining. Each dot represents one donor (n=3). Mean ± SD; paired t-test, two-tailed, relative to untreated cells. **(D)** CD8^+^ T lymphocytes treated as in **(C)** were used to extract RNA and measure *IL6* expression by qRT-PCR. Each dot represents one donor (n=3). Mean ± SD. * p < 0.05; **p < 0.01; ***p < 0.001; ****p < 0.0001.

Selected genes from this Nanostring dataset were validated using memory T cells from an independent set of donors. We found that *CSF2* expression was significantly reduced by vitamin D, while IFN-β alone was less effective (**Figure 4A**). IFN-β, alone or in combination with vitamin D, was instead a potent inducer of *IL10* expression. The ability of IFN-β to induce *IL17A* transcription was also confirmed on an independent set of donors, although this did not reflect in an increase in the percentage of cells able to produce IL-17 (**Supplementary Figure 1C**), suggesting that increased mRNA expression occurs only in the few circulating T lymphocytes that are already capable of secreting IL-17. *IL6* transcription was instead strongly increased by vitamin D, validating our Nanostring data and potentially correlating with the fact that the vitamin D receptor (VDR) was shown to directly bind the *IL6* locus, at least in DCs (30). Because the treatments also induced the expression of some markers of regulatory T (Treg) cells, such as *FOXP3*, we compared the expression of this gene in treated and untreated memory T cells, and also in freshly sorted Treg cells. We found that FOXP3 expression in treated memory T cells was overall much lower compared to Treg cells, and only modestly induced at protein level by vitamin D, arguing against any substantial Treg cell expansion or differentiation upon treatment, at least *in vitro* (**Figure 4B**).

Because CD8^+^ T lymphocytes are also important players in the pathogenesis of MS (1), we assessed the effect of IFN-β and vitamin D treatments on cytokine expression by memory CD8^+^ T lymphocytes. Similar to CD4^+^ cells, GM-CSF expression was significantly reduced in treated CD8^+^ T cells, although the predominant effect appeared to be linked to IFN-β rather than vitamin D (**Figure 4C** **)**. Similar to CD4^+^ T cells, IFN-β treatment (alone or in combination with vitamin D) induced IL-10 expression (**Figure 4C**), while transcription of the *IL6* gene was potently induced by vitamin D (**Figure 4D**). Overall, our data identified selected inflammatory cytokines and factors that are modulated by IFN-β and vitamin D, alone or in combination, acting directly on memory T lymphocytes.

### Vitamin D and IFN-β Modulate Gene Expression in Memory T Lymphocytes From Multiple Sclerosis Patients

**Figure 5:**
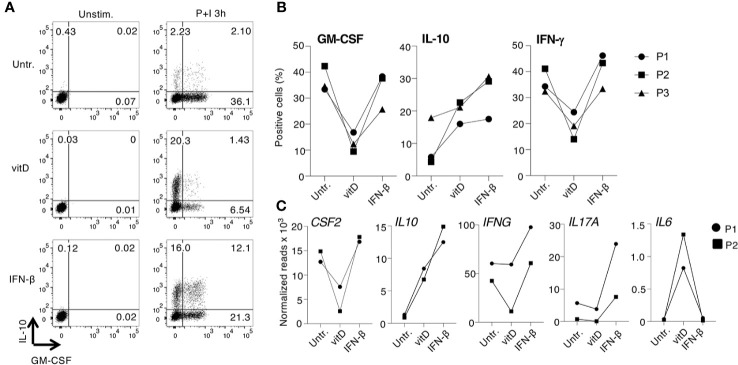
Expression of cytokines and immune transcripts in CD4^+^ T cells from multiple sclerosis (MS) patients. **(A)** Memory CD4^+^ T lymphocytes were freshly separated from the peripheral blood of one patient with MS at relapse, activated with plate-bound anti-CD3 and anti-CD28 antibodies and cultured for 5 days with vitamin D or IFN-β. Expression of IL-10 and GM-CSF was determined by intracellular staining. Representative of n=3 independent donors. **(B)** Cells from three independent MS patients were treated as in **(A)** and the percentages of GM-CSF, IL-10, and IFN-γ-producing cells was determined by intracellular staining (n=3). **(C)** Memory T cells obtained from two patients with MS were treated as in **(A)**, except that total RNA was extracted and the expression of ~570 immune-related genes was measured by Nanostring profiling. Results for selected transcripts are shown. The entire list of genes (including also data from naïve T cells from the same patients) can be found in **Supplementary Data Set 1** (n=2).

To assess the impact of IFN-β and vitamin D treatments on T cells from patients with MS, we separated memory T lymphocytes from three MS patients at the time of relapse. None of the patients was receiving any disease-modifying treatment. By intracellular staining, we found that vitamin D was remarkably effective at reducing GM-CSF and also IFN-γ expression in these CD4^+^ T cells, while IFN-β was much less efficient (**Figures 5A, B**). Similar to CD4^+^ T cells from healthy donors, IFN-β strongly induced IL-10 production. These results were comparable to the effects observed in T cells from healthy donors, suggesting intrinsic similar abilities of these cells to respond to the treatments, at least *in vitro*. To gain a broader understanding of the ability of MS patient-derived T cells to respond to treatments, we separated memory and naïve T lymphocytes from two MS patients at relapse. Cells were treated with either IFN-β or vitamin D and gene expression profiling was performed by Nanostring (**Figure 5C** and **Supplementary Data Set 1**). Overall, we observed responses consistent with those of T cells from healthy donors, namely reduced *CSF2* expression upon vitamin D treatment of memory T cells, increased *IL10* especially in response to IFN-β, a modest and variable reduction in *IFNG* expression, increased *IL17A* transcription in response to IFN-β, and increased *IL6* in response to vitamin D (**Figure 5C**).

### MicroRNA Expression Is Affected by Vitamin D and IFN-β Treatments

**Figure 6:**
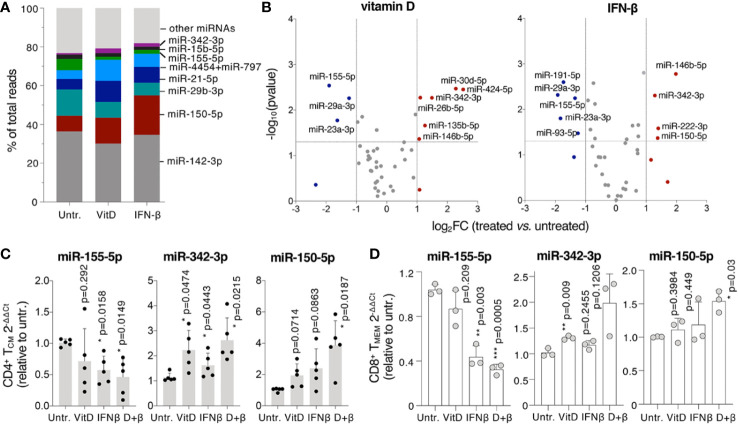
MicroRNA (MiRNA) expression is modulated by vitamin D and IFN-β. **(A)** T_CM_ cells were treated for 5 days in the presence or absence of vitamin D or IFN-β and miRNA expression was measured by Nanostring profiling (n=3 donors). The percentage of normalized reads for the indicated miRNAs is shown. **(B)** Differentially expressed miRNAs in the same samples as **(A)**. **(C)** T_CM_ cells were treated as in a) and the expression of selected miRNAs was measured by qRT-PCR. Each dot represents one donor (n=5). Mean ± SD; paired t-test, two-tailed, relative to untreated cells. **(D)** CD8^+^ memory T lymphocytes were activated and treated for 5 days, after which total RNA was extracted and the expression of the indicated miRNAs determined by qRT-PCR. Each dot represents one donor (n=3). Mean ± SD; paired t-test, two-tailed, relative to untreated cells.

MiRNAs regulate all aspects of T cell biology (31) and may mediate at least some of the effects of vitamin D and IFN-β treatment in modulating T cell responses. We therefore used digital counting to measure the expression of ~800 miRNAs in treated and untreated primary human T_CM_ cells (**Supplementary Data Set 1**). First, we found that a few miRNA species made up most of the miRNA content of T lymphocytes, with miR-142, miR-150, and miR-29b representing together more than 50% of the total miRNA reads, regardless of the treatment (**Figure 6A**). Next, we analyzed which miRNAs were significantly up- or down-regulated in response to treatments (**Figure 6B**). We found that vitamin D led to reduced expression of miR-155, a pro-inflammatory miRNA known to promote autoimmune inflammation (32), as well as of miR-29a and miR-23a, which are both known to impact T cell differentiation, proliferation and function (33, 34). Interestingly, miR-146b was modestly upregulated. MiR-146b shares the same “seed” sequence, and therefore the same predicted targets, with miR-146a, a miRNA strongly implicated in restraining inflammatory responses (35). Other upregulated miRNAs included miR-342, miR-30d, miR-26b, and miR-135b, all potentially involved in the regulation of cell proliferation, at least as reported in various, mostly non-immune cellular models (36, 37).

Similar to vitamin D, IFN-β reduced levels of miR-155, miR-29a and miR-23a and induced miR-146b and miR-342 expression. IFN-β also modestly induced the expression of miR-222 and miR-150, miRNAs known to regulate cell proliferation (38, 39). To assess the effect of the double vitamin D + IFN-β treatment we measured the expression of a few of the most differentially expressed miRNAs by qRT-PCRs, including miR-342 and miR-155, which were respectively up- or down-regulated by both treatments, and miR-150, which was significantly induced only by IFN-β (**Figure 6B**). In all cases the effect of the combined treatment was more pronounced compared to either vitamin D or IFN-β alone, although at different extents (**Figure 6C**). Similar effects on miRNA expression were observed in memory CD8^+^ T lymphocytes treated with vitamin D and IFN-β (**Figure 6D**). These differentially expressed miRNAs may therefore significantly contribute to modulate T cell biology in response to individual, and even more so combined, vitamin D and IFN-β treatment.

### T Cell Proliferation Is Significantly Affected by the Combined Vitamin D+IFN-β Treatment

**Figure 7:**
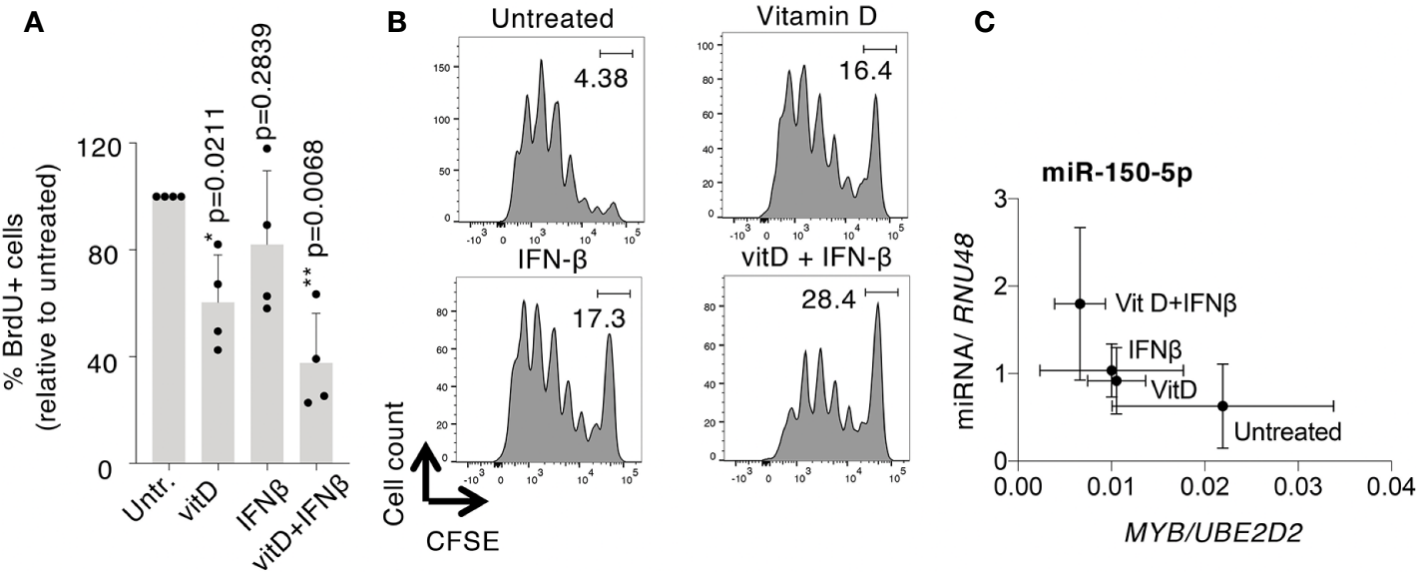
Vitamin D and IFN-β affect human T cell proliferation. T_CM_ cells were treated with vitamin D and/or IFN-β, followed by measurement of cell proliferation by **(A)** BrdU incorporation [each dot represents one donor (n=4), mean ± SD; paired t-test], or **(B)** CFSE dilution (representative of n=3 independent experiments). **(C)** Correlation of mean miR-150 and *MYB* expression (relative to endogenous controls) across the different treatments. Mean ± SD, n=5 independent donors.

Several of the differentially expressed miRNAs upon vitamin D and/or IFN-β treatment have confirmed or putative roles in regulating cell proliferation. By measuring cell proliferation in treated T cells, we found that vitamin D led to a significant reduction in BrdU incorporation and CFSE dilution, which was even more pronounced when the two treatments were applied at the same time, while IFN-β treatment alone had a more variable effect (**Figures 7A, B**). The suppression of T cell proliferation represents therefore one potential mechanism important to restrain inflammatory responses in treated cells.

Among the miRNAs potentially involved in the regulation of cell proliferation, miR-150 stood out because it is expressed at very high levels in T cells and because it was significantly induced by the combined treatments (**Figures 6A–D**). One of the established targets of miR-150 is the transcription factor MYB, which is directly involved in the regulation of T cell proliferation (40). *MYB* mRNA expression was indeed strongly reduced by the combined treatment with vitamin D+IFN-β, and anti-correlated with miR-150 expression. Specifically, we found that the combined treatment led to the highest levels of miR-150 expression, correlating with the lowest expression of *MYB*, compared to untreated cells, and also to cells that underwent treatment with only vitamin D or IFN-β (**Figure 7C**). Overall, our data point toward a potential regulatory module of cell proliferation that may be downstream of vitamin D and/or IFN-β signaling.

## Discussion

Here, we found that the treatment of primary human T lymphocytes with IFN-β and vitamin D led to complex transcriptomic changes that influenced primarily the expression of cytokines and miRNAs such as IL-10, GM-CSF, IL-6, miR-155, miR-342, and miR-150, as well as cell proliferation. Although the effects of the two treatments were largely distinguishable, their combined use led to significant synergy in the induction of an anti-inflammatory gene expression profile, characterized by low GM-CSF and miR-155 expression and high IL-10. IFN-β in particular was a potent inducer of IL-10 expression. There is a complex interplay between type I interferon signaling, activation of IRF transcription factors, cytokine expression, and miRNA regulation (41). For instance, IRF factors were shown to regulate directly the expression of the *IL10* gene (42), and at least in CD8^+^ T cells, miR-155 is a negative regulator of interferon signaling components required to induce optimal T cell responses. In particular, miR-155 deletion enhanced interferon signaling, impaired the capacity of T cells to mount primary and secondary responses and limited T cell proliferation (43). Similarly, mice lacking miR-155 showed defective inflammatory CD4^+^ T cell responses, and were resistant to EAE induction (32). In our study, we observed a significant downregulation of miR-155 expression in both CD8^+^ and CD4^+^ T lymphocytes upon treatment with vitamin D and IFN-β, that is therefore likely to contribute to diminished T cell responses. Such an effect would be potentially beneficial for MS patients. Interestingly, we also found that *CD274* (PD-L1) is a putative target of miR-155 [as predicted by TargetScan (44)] that was increased in all treated cells (**Figure 3A**), in which miR-155 was instead downregulated. *CD274* is an interferon-regulated gene (45), therefore its increased expression is likely to derive from a combination of increased transcription and reduced miRNA targeting. Because of the crucial importance of PD-1:PD-L1 in the regulation of T cell tolerance and in autoimmune diseases (45), modulation of this pathway may also be beneficial in autoimmunity (46). As for the putative miR-342 and miR-150 targets, no other relevant gene was identified in the intersection of predicted targets with the list of downregulated genes, most likely due to the limited number of genes that were downregulated upon treatment. Interestingly, increased miR-342 expression in Treg cells was recently shown to enhance Treg cell suppressive functions and to attenuate EAE in mice, pointing toward a beneficial role of this miRNA in MS (47).

Whether IRF transcription factors (or VDR) also directly regulate the promoter activity of other affected genes and miRNAs remains to be established. In the mouse, *VDR*-deficiency specifically in CD4^+^ T cells was necessary for vitamin D to ameliorate EAE induction (48), pointing toward a direct role VDR engagement in the regulation of CD4^+^ T cell functions. Interestingly, we recently found that *VDR* gene expression was associated with high levels of GM-CSF expression in primary human T lymphocytes (49), suggesting that T cells with the highest inflammatory potential may also be the ones that are more likely to be regulated by vitamin D, a further mechanism of T cell modulation that could be beneficial in autoimmunity. Finally, a genome-wide analysis of VDR binding sites identified the *IRF8* locus as directly bound by this transcription factor, which may further contribute to the combined effect of vitamin D and IFN-β treatment on gene expression (27). As for the differential miRNA expression, none of the identified miRNAs target directly cytokine genes, and they are therefore likely to influence the general inflammatory potential of T lymphocytes, for instance by regulating NF-kB activation (e.g., miR-146 family members) or cell proliferation (e.g., miR-150 and miR-155).

While we focused primarily on the responses of CD4^+^ T helper lymphocytes, it is becoming increasingly clear that also CD8^+^ T cells and other immune cell types, such as B lymphocytes and innate immune cells, play a key role in the development or severity of MS (1, 6). Though we described some of the direct effects exerted by IFN-β and vitamin D treatments on gene and miRNA expression in CD4^+^ and CD8^+^ T lymphocytes, other direct and indirect effects may undoubtedly affect lymphocytes’ responses *in vivo.* Indeed, both IFN-β and vitamin D have widespread effects on a number of cell types and systems. For example, IFN-β treatment of DCs led to reduced T_H_17 polarization *in vitro* (50), suggesting that *in vivo* the final outcome on the T cell phenotype following IFN-β treatment depends on both direct effects on T cells and indirect effects on other cell types, including reduced expression of DC-derived cytokines that regulate CD4^+^ T cell differentiation (9, 10). Moreover, the IFN-α/β receptor (IFNAR) is ubiquitously and constitutively expressed (51). Indeed, while our study revealed a direct anti-inflammatory effect of IFN-β on memory T lymphocytes, mice broadly devoid of IFNAR developed exacerbated EAE, linked most prominently to dysregulated myeloid cell functions (12). The interplay of direct and indirect effects in the case of vitamin D is also complex, since virtually all immune cells express the VDR, and many immune cell types, including T lymphocytes, can convert vitamin D into its active form (52).

Inflammatory cytokines are important factors in deciding the fate of activated naive cells. In particular, cytokines such as IL-6 and IL-1β, in combination with TGF-β, may lead to enhanced differentiation toward the pathogenic T_H_17 subset (53). In this regard, the increased IL-6 production observed with vitamin D treatment may also influence T cell differentiation to a phenotype that would be undesired in the context of autoimmunity. For instance, levels of both IL-17 and IL-6 were shown to positively correlate with neurological disabilities in MS patients (18), and blockade of IL-6-dependent pathways using an anti-IL-6 receptor antibody limited the development of EAE in mouse models, most likely by counteracting the differentiation and expansion of pathogenic T_H_17 cells while promoting Treg cell responses (54, 55). The composite effect of vitamin D on T lymphocytes, namely its ability to downmodulate pro-inflammatory cytokines such as GM-CSF, while at the same time inducing IL-6 expression, may complicate our understanding of its mechanism of action *in vivo*, and may at least in part contribute to the limited effect observed in clinical trials using high doses of vitamin D. These observations warrant additional studies to fully understand the role of vitamin D and T cell-derived IL-6 in chronic inflammatory diseases.

## Data Availability Statement

The datasets presented in this study can be found in the **Supplementary Material** for this article.

## Ethics Statement

The studies involving human participants were reviewed and approved by Comitato Etico Canton Ticino, authorization number CE 3428 and CE TI 2906. The patients/participants provided their written informed consent to participate in this study.

## Author Contributions

NB and SE designed and performed experiments, analyzed data, and wrote the manuscript. CZ provided MS patients’ samples and wrote the manuscript. SM overviewed the study, analyzed data, and wrote the manuscript. All authors contributed to the article and approved the submitted version.

## Funding

This work was funded by the NCCR “RNA & Disease”, the Swiss MS Society, the Vontobel-Stiftung, the Ceresio Foundation and the Swiss National Science Foundation (grant number 175569), all to SM.

## Conflict of Interest

CZ received honoraria for speaking, consulting fees, or research grants from Abbvie, Almirall, Biogen Idec, Bayer, Celgene, Genzyme, Merck Serono, Novartis, Teva Pharma, Roche.

The remaining authors declare that the research was conducted in the absence of any commercial or financial relationships that could be construed as a potential conflict of interest.
