# Supplementary material for: Vitamin D and IFN-β Modulate the Inflammatory Gene Expression Program of Primary Human T Lymphocytes
Source: Front Immunol. 2020 Dec 4;11:566781. doi: 10.3389/fimmu.2020.566781 (PMC7746617; doi:10.3389/fimmu.2020.566781)
Supplement: Supplementary file 2 [file Table_1.docx]

Supplementary Material

**Vitamin D and IFN-β modulate the inflammatory gene expression program of primary human T lymphocytes**

Bianchi, Emming *et al.*

**Supplementary Figure 1.** **a)** Gating strategy for naïve and memory CD4^+^ and CD8^+^ T lymphocyte separation. **b)** T_N_ and T_EM_ cells from different donors were treated as in Figure 1a, and the percentage of GM-CSF^+^ and IL-10^+^ cells was determined by intracellular staining. Each dot represents one donor (at least n=3). Mean ± SD; unpaired t-test, two-tailed, relative to untreated cells. **c)** Memory CD4^+^ T cells were activated with plate-bound anti-CD3 and anti-CD28 antibodies in the presence or absence of vitamin D and/ or IFN-β. Intracellular staining for IFN-γ and IL-17 expression was performed after 5 days of culture using conjugated antibodies (IL-17A-APC, IFN-γ-APC-Cy7). Each dot represents one donor (n=5). Mean ± SD; paired t-test, two-tailed, relative to untreated cells.

**Supplementary Table 1. Reagents used in this study.**

**Treatments**

|  | **Company** | **Catalog number** |
| --- | --- | --- |
| 1α,25-Dihydroxyvitamin D3 | Sigma | D1530-10UG |
| human IFN-β | Peprotech | 300-02BC |

**Antibodies**

| **Antibody** | **Company** | **Catalog number** |
| --- | --- | --- |
| αGM-CSF (PerCp/Cy5.5) | Biolegend | 502311 |
| αIL-17A (bv605) | Biolegend | 512325 |
| αIFN-γ (APC/Cy7) | Biolegend | 502530 |
| αIL-10 (bv421) | Biolegend | 501421 |
| αCD4 (PE-TR) | Invitrogen | MHCD0417 |
| αCD25 (PC5) | Beckman Coulter | IM2646 |
| αCD45RA (QD655) | Invitrogen | Q10069 |
| αCCR7 (bv421) | Biolegend | 353208 |
| αCD3 (TR66) | Recombinant antibody, provided by in-house facility | |
| αCD28 | BD Pharmingen | 555725 |

**Primers for SYBR qPCR**

| **Target gene** | **Primer fw** | **Primer rv** |
| --- | --- | --- |
| *CSF2* | 5′-TGTGAATGCCATCCAGGAGG | 5′-GCTTGTACAGCTCCAGGCG |
| *IL10* | 5′-CCAAGACCCAGACATCAAGG | 5′- GGCCTTGCTCTTGTTTTCAC |
| *IFNG* | 5′-CGAGATGACTTCGAAAAGCTG | 5′-CAGTTCAGCCATCACTTGGA |
| *IL17A* | 5′-CCACCTCACCTTGGAATCTC | 5′-TGGTAGTCCACGTTCCCATC |
| *IL6* | 5′-AACAACCTGAACCTTCCAAAGA | 5′-TCAAACTCCAAAAGACCAGTGA |
| *FOXP3* | 5′-CAAATGGTGTCTGCAAGTGG | 5′-TGCCCTTCTCATCCAGAAGAT |
| *MYB* | 5′-GCATAACCACTTGAATCCAGAAG | 5′-ATTATCAGTTCGTCCAGGCAGTA |
|  |  |  |
| **Endogenous control** |  |  |
| *UBE2D2* | 5′-GATCACAGTGGTCTCCAGCA | 5′-CGAGCAATCTCAGGCACTAA |

**Taqman probes**

| **Target miRNAs** |  |
| --- | --- |
| miR-155-5p | Hs002623 |
| miR-342-3p | Hs002260 |
| miR-150-5p | Hs000473 |
|  |  |
| **Endogenous control** |  |
| RNU48 | Hs001006 |
